# GLORB: Robust Bayesian inference for differential expression under global expression shifts

**DOI:** 10.64898/2026.08.28.747928

**Published:** 2026-09-02

**Authors:** Rowan Callahan, Stephen D. Coleman, Thuy T. M. Ngo

## Abstract

Estimating differential gene expression is a common task in RNA-seq. Current methods mostly rely on normalization to reduce variance and increase accuracy. These methods are widely used and provide invaluable information about transcriptomic changes between biological conditions. However, widely used normalization methods are known to distort estimates of transcript differential expression when a majority of genes are upregulated or downregulated, or when the total RNA content per cell changes. Despite the presence of global expression shifts in a number of contexts, few methods exist that can provide accurate normalization and estimate linear models under this context without spike-in controls. Here, we present **GLORB** ( **GLO** bal-shift **R** obust **B** ayesian model), a method for estimating generalized linear models under global upregulation. We develop two models that are able to recover differentially expressed genes and linear model coefficients with lower distortion of results. We show that our model’s method of accounting for library size variance is consistent with DESeq2’s median of ratios, and edgeR’s trimmed mean of M-values under conditions when a minority of genes are upregulated and outperforms them under circumstances when most genes are either increased or decreased between groups. Finally, because our method does not rely on calculating geometric means for each gene it is able to work in datasets with much higher sparsity.

## Full Text Availability

The license terms selected by the author(s) for this preprint version do not permit archiving in PMC. The full text is available from the preprint server.

